# Glycated Hemoglobin as a Predictor of the Length of Hospital Stay in Patients Following Coronary Bypass Graft Surgery in the Saudi Population

**DOI:** 10.21470/1678-9741-2018-0202

**Published:** 2019

**Authors:** Joud G. Almogati, Elnazeer O. Ahmed

**Affiliations:** 1 Faculty of Medicine, King Abdulaziz University, Kingdom of Saudi Arabia.; 2 Cardiac Surgery Department, King Abdullah Medical City, Makkah, Kingdom of Saudi Arabia.

**Keywords:** Myocardial Revascularization, Length of Stay, Glycated Hemoglobin

## Abstract

**Objective:**

The diabetic population has a high prevalence of coronary artery disease, and frequently patients with diabetes undergo coronary artery bypass graft (CABG) surgery. Elevated glycated hemoglobin (HbA1c) in diabetics is shown to be associated with morbidity and mortality, but the association of HbA1c with postoperative length of hospital stay (LOS) has conflicting results. In this study, we aim to identify if elevated HbA1c levels are associated with prolonged LOS after CABG surgery.

**Methods:**

A retrospective chart review study was performed, using a total of 305 patients who were referred for CABG surgery. HbA1c levels were measured before the day of surgery. Patients were classified into two groups according to HbA1c levels: <7% and ≥7%. A LOS of more than 14 days was proposed as an extended LOS. HbA1c and the LOS relationship were assessed using appropriate statistical methods.

**Results:**

Patients who had diabetes mellitus comprised 81.6% of our studied population. Sixty-four percent had HbA1c levels ≥ 7%. There was no significant difference in the total LOS in HbA1c <7% compared to HbA1c ≥7% patients (*P*=0.367).

**Conclusion:**

Our study results rejected the proposed hypothesis that elevated HbA1c levels ≥7% would be associated with prolonged hospital stay following CABG surgery in a Saudi population.

**Table:** 

Abbreviations, acronyms & symbols	
CABG	= Coronary artery bypass graft
HbA1c	= Hemoglobin A1c or glycated hemoglobin
HPLC	= High pressure liquid chromatography
ICU	= Intensive care unit
LOS	= Length of hospital stay
LVEF	= Left ventricular ejection fraction
NYHA	= New York Heart Association
SD	= Standard deviation
STS	= Society of Thoracic Surgeons

## INTRODUCTION

There are 425 million people with diabetes in the world, and this prevalence is estimated to reach 629 million in the year of 2045^[1]^. The reported prevalence of diabetes in Saudi Arabia is high, at about 30%^[2]^. Overall, vascular diseases, which include coronary heart disease and cerebrovascular disease, account for 65% of all deaths among diabetic patients^[3]^, and this mortality is related to the baseline HbA1c level^[4-6]^.

Plasma hemoglobin A1c (HbA1c) reflects mean ambient fasting and postprandial glycemia over the preceding 2-3 months. It is formed by the slow irreversible, non-enzymatic glycation of valine and lysine residues in the hemoglobin molecule^[7]^.

Coronary heart disease is common in the diabetic population, and frequently patients with diabetes need coronary artery bypass graft (CABG) surgery^[8]^. Within the United States alone, approximately 500,000 patients undergo CABG surgery annually, of whom 20% have diabetes mellitus^[9-11]^. The reported incidence in Saudi Arabia of CABG in patients who have diabetes is 61.2% and 78.8% for males and females, respectively^[12]^.

Prevalence of high HbA1c is common among patients undergoing CABG surgery^[13,14]^, and it was found to be a risk marker for this surgery^[15]^. Additionally, elevated HbA1c levels preceding CABG surgery are strongly related to the severity of subsequent complications, such as in-hospital death, renal failure^[9,16]^, myocardial infarction, cerebrovascular accident, and deep sternal wound infection^[16,17]^. Hyperglycemia before surgery was found to be associated with an increased risk for complications after surgery. Additionally, controlling hyperglycemia after cardiac surgery was shown to decrease the rate of deep sternal wound infection^[18]^, myocardial ischemia, and other complications^[19]^.

Previous reports about the influence of poor long-term glycemic control on postoperative length of hospital stay (LOS) have conflicting results^[17,20-24]^.

Our study aims to establish if HbA1c levels can predict LOS in diabetic patients after CABG surgery.

## METHODS

A retrospective chart review study was performed, using 315 patients who were referred for CABG surgery at King Abdullah medical city (Makkah, Saudi Arabia) from November 2013 to May 2015. Of the 315 patients, 10 patients had incomplete data, such as missing HbA1c levels, and they were not included in the study. The final population consisted of 305 patients, and the cardiac surgery department database was used to retrieve patient data. The Institutional Review Board approved the study and waved the need for individual patient consent.

The clinical data included in our study were: age, sex, body mass index, New York Heart Association (NYHA) functional class, number of diseased vessels, and left ventricular ejection fraction (LVEF). The patients' history of diabetes mellitus, hypertension, dyslipidemia, history of peripheral vascular disease, and post-operative complications was also noted.

HbA1c levels were measured on the day of surgery or within 3 days before the operation using high pressure liquid chromatography (HPLC). Patients were classified into the following two groups based on their HbA1c levels: <7% and ≥7%. A HbA1c level ≥7% was used as the threshold for uncontrolled hyperglycemia, because this level is recognized by the American Diabetes Association as representing poor diabetes control^[25]^. A LOS of more than 14 days was proposed as an extended LOS, as defined by the Society of Thoracic Surgeons (STS)^[26]^.

### Statistical Analysis

Categorized variables were presented as percentages and frequencies, while the quantitative variables are given as the mean ± standard deviation (SD). The relationship between HbA1c and LOS was assessed using Pearson's Chi-square test. Clinical characteristics and morbidities were analyzed using an independent t-test and Pearson's Chi-square test where appropriate to determine the predictors of LOS. The variables were entered into a logistic regression model according to their statistical significance.

SPSS Version 16 for Windows (SPSS, Inc., Chicago, IL, USA) was used for statistical analysis. A *P*-value of <0.05 was assumed to represent statistical significance.

## RESULTS

The clinical characteristics and preoperative data for patients are presented in Table 1. Patients who had diabetes mellitus comprised 81.6% (249/305) of our studied population, with a mean age 59.1 years (range, 31-87 years). The range of HbA1c was 4.9%-15%, and 64% (195/305) had HbA1c levels ≥7%. Of those patients, 96.4% (188/195) were known to have diabetes.

**Table 1:** Clinical characteristics of CABG patients (all patients n=305).

Clinical characteristics		Results
Mean age (year), (SD)		59.10 (± 9.67)
Male sex, n (%)		250 (82)
Mean body mass index (kg/m^2^), (SD)		27.80 (±5.88)
Diabetes mellitus, n (%)		249 (81.6)
Hypertension, n (%)		256 (83.9)
Dyslipidemia, n (%)		143 (46.9)
History of PVD, n (%)		9 (3)
NYHA functional class, n (%)	1	2(0.7)
	2	164 (53.4)
	3	109 (35.7)
	4	30 (9.8)
Number of diseased vessels, n (%)	1	3 (1)
	2	32 (10.5)
	3	270 (88.5)
Left ventricular ejection fraction % mean (range)		43.7 (15-68)
Patients with HbA1c ≥7%, n (%)		195 (63.9)
Mean HbA1c, (SD)		8.2 (±2.16)
Mean EuroSCORE, (SD)		4.3 (±4.25)

HbA1c=glycated hemoglobin; NYHA=New York Heart Association; PVD=peripheral vascular disease; SD=standard deviation

Complications are presented in Table 2. A total of 100 patients out of 305 (32.78%) had postoperative complications within 30 days. The most frequent complications were atrial fibrillation (31.85%, 97/305) and renal failure (5.2%, 16/305). Twelve (3.93%) patients died, and their mean EuroSCORE was 8.82 (range, 1.05 to 26.24). Twelve (3.9%) patients developed mediastinitis, and nine of them were diabetic. Postoperative LOS ranged from 2 to 90 days (mean, 11.77 ± 11.86 days), with a median of 8 days. Sixty-seven percent of patients (204/305) had a LOS ≤10 days, 21% of patients (64/305) had LOS ≥14 days, and only 3.6% of patients (11/305) had LOS ≥40 days.

**Table 2:** Postoperative morbidity and mortality (all patients n=305).

Events	Number	Percent (%)
Postoperative deaths	12	3.93
Atrial fibrillation	97	31.8
Cerebrovascular accident	6	2
Renal impairment	16	5.2
Surgical site infection	12	3.9

Table 3 presents surgical outcomes related to preoperative HbA1c levels (with HbA1c <7% compared to HbA1c ≥7%). There is no correlation between increased HbA1c levels and 30-day mortality rates, arrhythmia, cerebrovascular accident, renal failure requiring dialysis, or mediastinitis.

**Table 3:** Comparison of postoperative morbidity and mortality with preoperative HbA1c levels.

Variables	HbA1c <7% (n=110)	HbA1c ≥7% (n=195)	*P* value
Postoperative deaths	3 (0.9%)	12 (6.2%)	0.409
Arrhythmia	30 (27.3%)	67 (34.4%)	0.202
Cerebrovascular accident	1 (0.9%)	5 (0.5%)	0.600
Renal failure requiring dialysis	5 (4.5%)	12 (6.2%)	0.680
Mediastinitis	3 (0.9%)	9 (2.9%)	0.415

Level of significance, *P*<0.05. HbA1c=glycated hemoglobin

Table 4 presents the predictors of CABG surgery LOS, and there was no significant difference in HbA1c <7% compared to HbA1c ≥7 for total LOS using Pearson's Chi-square test *(P*=0.367). Using the independent t-test, LVEF% was the only predictor of LOS to reach statistical significance (*P*=0.001). Additionally, the logistic regression analysis for LVEF% and LOS (<14 days, ≥14 days) was significant (*P*≤0.001).

**Table 4:** Predictors of length of hospital stay post coronary artery bypass graft surgery.

Variables		LOS < 14 days (n=241)	LOS ≥ 14 days (n=64)	*P* value
Age		59.1 ±9.7	58.9 ±9.3	0.856
Male sex		200 (74)	50 (78)	0.368
Diabetes mellitus		196 (81.3)	53 (82.8)	0.785
Hypertension		203 (84.2)	53 (82.8)	0.783
Dyslipidemia		111 (46)	32 (50)	0.574
PVD history		6 (2.5)	3 (4.6)	0.356
NYHA functional class	1	2 (0.8)	0	0.724
	2	129 (53.5)	35 (54.6)	
	3	88 (36.5)	21 (32.8)	
	4	22 (9.1)	8 (12.5)	
Number of diseased vessels	1	3 (1.2)	0	0.665
	2	25 (10.3)	7 (10.9)	
	3	213 (88.4)	57 (89)	
LVEF %		44.8 ±9.3	39.6 ±9.9	<0.001
HbA1c	< 7%	90 (37.3)	20 (31.3)	0.367
	≥ 7%	151 (62.6)	44 (68.8)	

Data are presented as the mean ±SD or n (%) HbA1c=glycated hemoglobin; LOS=Length of hospital stay; LVEF=left ventricular ejection fraction; NYHA = New York Heart Association; PVD=peripheral vascular disease

## DISCUSSION

In this study of 305 patients, we investigated as to whether those with elevated HbA1c levels had a longer LOS compared with those with optimal HbA1c levels CABG. We found that LOS was not affected by preoperative HbA1c levels (with a cut-off point of HbA1c = 7% for optimal and suboptimal levels; *P*=0.367). This is in agreement with the results of previous studies that investigated the above relationship^[21,22]^. Knapik et al.^[20]^ reported a study in 2010 with a population of 782 diabetic patients, which showed no significant difference in total LOS (with HbA1c ≤7% compared to HbA1c >7%; *P*=0.59). Matsuura et al.^[21]^ reported similar results in a study in 2009.

Conversely, Medhi et al.^[23]^ conducted a study in 2001 with 135 patients, showing that HbA1c was significantly associated with postoperative LOS *(P*=0.025), while in a 2012 study by Najafi et al.^[22]^, similar results were found (*P*=0.01). These disparities may be a result of different cut-off points to define a prolonged hospital stay (from ≥3 to 7 days)^[20,22,23]^, different sample sizes, and different study populations.

Postoperative LOS ranged from 2 to 90 days (mean, 11.77 ± 11.86 days) with a median of 8 days. This is longer than the post-CABG LOS that was reported by others^[22,23]^.

We found that the LVEF can predict LOS following CABG (*P*=0.001). Najafi et al. ^[22]^ reported that LVEF was not a significant predictor of LOS, but their findings were only for intensive care unit (ICU) LOS, and not for the overall length of hospital stay. The most common complication was atrial fibrillation, which was observed in about 32% of patients. This is similar to results reported in previous studies^[22,23]^. There was no significant difference in the post-operative complication rate between HbA1c <7% and HbA1c ≥7%. A study in 2017 by Finger et al.^[17]^ showed similar results, except that sternal wound infections were significantly increased in the HbA1c ≥7% group (*P*=0.030).

In diabetics scheduled for CABG, poor preoperative glycemic control (suggested by HbA1c) is common, and 64% of our population had HbA1c ≥7%.

To our knowledge, this study is the first to present data on the prevalence of elevated HbA1c in patients undergoing CABG, and its relation to LOS in Saudi Arabia.

The limitations of this study were that it was conducted at one health center, which may limit its generalizability, and its retrospective nature; not all the information was available as a result of poor documentation because the data was recorded in the past. However, there was a sufficiently large sample size for our purposes.

## CONCLUSION

Our study results rejected the proposed hypothesis that elevated HbA1c levels >7% would be associated with a prolonged hospital stay following CABG surgery in a Saudi population.

**Table:** 

Authors' roles & responsibilities	
JGA	Contributions to the design, acquisition, interpretation of data and drafting the work; final approval of the version to be published
EOA	Contributions to the design, acquisition, interpretation of data and drafting the work; final approval of the version to be published
